# Interim efficacy and safety of PD-1 inhibitors in preventing recurrence of hepatocellular carcinoma after interventional therapy

**DOI:** 10.3389/fimmu.2022.1019772

**Published:** 2022-10-28

**Authors:** Wenying Qiao, Qi Wang, Caixia Hu, Yinghua Zhang, Jianjun Li, Yu Sun, Chunwang Yuan, Wen Wang, Biyu Liu, Yonghong Zhang

**Affiliations:** ^1^ Interventional Therapy Center for Oncology, Beijing You ‘an Hospital, Capital Medical University, Beijing, China; ^2^ Center for Infectious Diseases, Beijing You ‘an Hospital, Capital Medical University, Beijing, China; ^3^ Research Center for Biomedical Resources, Beijing You ‘an Hospital, Capital Medical University, Beijing, China

**Keywords:** hepatocellular carcinoma, PD-1 inhibitors, TACE, ablation, immune, recurrence

## Abstract

**Introduction:**

Locoregional interventional therapy including transcatheter arterial chemoembolization (TACE) and ablation are the current standard of treatment for early-to-mid-stage hepatocellular carcinoma (HCC). However, questions remain unanswered regarding the management of recurrence after locoregional treatment. PD-1 inhibitors can block inhibitory signals of T-cell activation and proliferation to reduce the recurrence. We conducted a single-arm phase 2 trial to evaluate the efficacy and safety of PD-1 inhibitors following locoregional interventional therapy in HCC patients with high recurrence risk guided by our novel scoring system.

**Methods:**

Patients enrolled initially treated by TACE combined with ablation, then willingly joined the experimental group. One month later, they received the anti-PD-1 adjuvant therapy (intravenous injection of 200 mg), which was repeated every 3 weeks for a total of 4 or 8 cycles. Within this same period, other patients were screened into the control group to match the experimental group by 1:1 based on the propensity score matching method (PSM). The primary endpoint was relapse-free survival (RFS). Secondary endpoints included overall survival (OS) recurrence modality, safety, and quality of life.

**Result:**

At the time of data cutoff, the median RFS of the control group was 7.0 months while the experimental group had not reached it. Moreover, the 1-year RFS rate was 73.3% in the experimental group and 46.7% in the control group, showing a significant difference (P =0.02). The rate of local tumor progression in the experimental group was clearly lower than that in the control group (P = 0.027). Benefits associated with anti-PD-1 adjuvant therapy were observed in patients with multiple tumors and tumor size ≤2cm. Univariate and multivariate analyses demonstrated that anti-PD-1 adjuvant therapy was an independent favorable prognostic factor for RFS in HCC patients. The most frequent AE observed in this study was RCCEP, and other AEs included diarrhea, hepatotoxicity, rash, pruritus, and fatigue. The incidence of GRADE ≥3 AE and withdrawal in this study was low with no deaths recorded.

**Conclusions:**

Interim analysis from the study suggest the addition of anti-PD-1 adjuvant therapy after TACE combined with ablation could significantly prolong RFS with controllable safety for early-to-mid-stage HCC patients with high recurrence risk.

## Introduction

Hepatocellular carcinoma (HCC) was the sixth most common tumor and had the third-highest cancer-related mortality worldwide in 2020 (1). Unlike other types of cancer, where surgery, radiation, and systemic therapies dominate the therapeutic landscape, in HCC loco-regional interventional treatments are the mainstay of therapeutic options (2–4). Ablation is one of the foundation options for early-stage HCC, with a 5-year survival rate of 70% (5), while transcatheter arterial chemoembolization (TACE) is the main treatment method for the intermediate stage, with an estimated survival time of over 2 years (6).

In the last decade, treatment modalities for different stages of tumors, such as targeted therapy, immunotherapy, and chemotherapy, have made tremendous progress which effectively enhances the prognosis of patients (7–10). However, questions remain unanswered regarding the management of recurrence after locoregional treatment. Some patients recurrent quite early after locoregional interventional therapies, the median time to recurrence is 20-30 months, even in patients at the early stage (11–13). Numerous scholars have devoted themselves to exploring solutions, focusing on two primary strategies. Identifying high-risk patients is one of the main approaches (14). Many factors could predict treatment failures, such as tumor size, alpha-fetoprotein (AFP), Child-Pugh score, and BCLC stages (15–18). Our team developed a novel scoring system based on gender, tumor number, AFP, Fib, and albumin-to-prealbumin ratio to stratify patients with HCC into groups with different recurrence risks (19). At one year after locoregional interventional therapy, the recurrence rate in the low and intermediate-risk groups was 4% and 23.4%, while the high-risk group was 47.3%, with an area under the curve of 0.68.

Adjuvant therapy following locoregional intervention therapies is the other potential solution to recurrence (20). The immunity role of T cells is known to play a critical role during tumorigenesis and development (21). Our team’s studies on tumor-specific T-cell immune responses in HCC patients showed that: compared with the advanced stage, HCC patients with early-stage had broad-spectrum immunity and high-intensity SMNMS (SALL4, MAGE-A3, NY-ESO-1, MAGE-A1, SSX2) specific T cell immune responses, which could delay tumor recurrence after ablation (22). Furthermore, the relapsed patients after ablation showed activation of the PD-1/PD-L1 pathway in PBMC methylation levels compared to non-relapsed patients, suggesting that the activation of PD-1/PD-L1 pathway was not conducive to the control of tumor by the immune system (unpublished data). Moreover, PD-1 inhibitors can block inhibitory signals of T-cell activation and proliferation so as to restore immune function (23).

Hence, we conducted a single-arm phase 2 trial to evaluate the efficacy and safety of PD-1 inhibitors following locoregional interventional therapy in HCC patients with high risk guided by our novel scoring system.

## Patients and methods

### Patients

Eligible patients were aged 18 to 75 years old and had a pathological or radiographic confirmed diagnosis of HCC that met the criteria of the American Association for the Study of Liver Diseases (24), with the goal of complete ablation which was defined as complete non-enhancement of treated tumor on contrast-enhanced computed tomography (CT). All patients classified as China liver cancer staging (CNLC) I a, I b, II a, or II b satisfied the criteria of Class A or B of the Child-Pugh classification and had an Eastern Cooperative Oncology Group (ECOG) performance status of 0 or 1. Only high relapse-risk patients were included, which was evaluated by a scoring system (19)established in our previous research.

The exclusion criteria were as follows (1): major surgery was performed within 3 weeks before treatment (2); other malignant diseases were diagnosed in the past 5 years (3); advanced HCC (4); autoimmune liver disease (5); received other therapies, such as Chinese patent medicine, drugs with immunomodulatory effects, glucocorticoid therapy or other immunosuppressive therapies (6); received anti-PD-1/PD-L1 therapy. Complete eligibility criteria are provided in the trial protocol.

The clinical trial was conducted according to the Declaration of Helsinki and the Good Clinical Practice guidelines of the International Conference of Harmonisation. The protocol and amendments were approved by the ethics committee of the Beijing You’an Hospital affiliated to Capital Medical University (Ethics approval number: 2020-118), and all patients signed written informed consent forms. This study was registered at the Chinese Clinical Trial Registry (ChiCTR2000038949).

### Trial designs and treatment

Patients enrolled initially treated by TACE combined with ablation (hereinafter referred to as combination therapy) and achieved complete remission which is defined as the presence of an ablative margin of at least 5 mm around the entire tumor, no more enhancing area in the arterial phase and no more defect in the portal phase on enhanced CT scan (25, 26). Then, patients willingly joined the clinical trial (experimental group) for anti-PD-1 adjuvant therapy. Within the same period, patients were screened into the control group from patients who disagreed to receive anti-PD-1 adjuvant therapy after having received combination therapy, based on the inclusion and exclusion criteria and the scoring system. Lastly, patients in two groups were 1:1 matched on the basis of the propensity score matching method (PSM), to ensure well-balanced significant variables between groups and make the groups comparable.

The TACE procedure was performed by two interventional radiologists with five years of experience with this approach. The right femoral artery was cannulated by percutaneous puncture under local anesthesia. The hepatic tube was delivered to the hepatic artery *via* an ultra-slip guidewire and connected to a high-pressure syringe under DSA with a total volume of 16 ml and a flow rate of 4 ml/s to visualize the intrinsic hepatic artery, right and left hepatic arteries and branches. A highly flexible coaxial microcatheter was delivered into the tumor-supplying artery using selective/super-selective techniques, after which the doxorubicin and lipiodol mixture was injected. And the microcatheter was connected to a high-pressure syringe for imaging. Finally, embolization materials, such as gelatin sponges or polyvinyl alcohol particles, were then used to embolize until complete stasis of the blood flow in the vessels. The doses of the drug were based on patients’ white blood cell count, platelet count, and liver function. Angiography showed intratumoral vessel occlusion, embolization agent filling, and tumor staining disappearance, which was considered the endpoint of embolization.

Local ablation was performed under the guidance of CT or magnetic resonance imaging (MRI) within 2 weeks after TACE. The procedures were summarized as follows (1): the appropriate location was selected by CT or MRI to determine the ablation procedure (2); after disinfection, spreading towels, and puncture site anaesthesia, the ablation needles were inserted into the skin (3); multiple overlapping ablations should be considered based on tumor size and tumor number, then timely image scanning to track the ablation process (4); after ablation, the ablation needle was pulled out and the needle track was ablated to prevent bleeding and metastasis. Regardless of the choice of single or fractional ablation, the safe ablation range of 0.5-1.0cm should be reserved to ensure complete coverage of the tumor and achieve complete ablation.

One month after ablation, patients in the experimental group received the anti-PD-1 adjuvant therapy (intravenous injection of 200 mg) repeated every 3 weeks for 4 or 8 cycles, according to clinical guidelines. The treatment was continued until disease progression, unacceptable toxicity, consent withdrawal, investigator decision, or receiving adequate treatment cycles, whichever occurred first.

If any adverse events (AEs) occurred during the trial, the possible reasons first needed to be determined as quickly as possible by the investigator. Then, the dose was adjusted depending on the severity of the AEs occurred in the previous dosing cycle.

### Endpoints and assessments

The primary endpoint was relapse-free survival (RFS) defined as the time from local interventional therapy to the time of recurrence or the follow-up deadline. The secondary endpoints included overall survival (OS), recurrence modality, safety, and quality of life. The OS is calculated from the date of initial treatment to the follow-up deadline or death.

The recurrence modality was classified as local tumor progression (LTP), intrahepatic distant recurrence (IDR), and extrahepatic metastasis (EM) based on recommendations by the International Working Group on Image-Guided Tumor Ablation (27). LTP was designated as tumor recurrence within or adjacent to the original ablation lesion (<2.0 cm from the edge of the ablation site). IDR was defined as a new tumor with typical HCC enhancement features within different liver subsegments distinct from the original ablation site. And extrahepatic metastasis (ED) was defined as metastases outside the liver.

Patients were scheduled to be followed up every 3 months. Recurrence as endpoints of interest was confirmed by contrast-enhanced CT or MRI, which were evaluated at baseline and every 3 months thereafter. Then the clinical examinations, including blood routine, liver biochemistry, AFP, coagulation test, and thyroid function tests, were recorded and laboratory assessments were undertaken before administration of each dose.

AEs were monitored and graded according to the National Cancer Institute (NCI) Common Terminology Criteria for Adverse Events (CTCAE) Version 5.0. After treatment, patients were followed up for safety for up to 30 days and for long-term survival to monitor AE.

Patient’s quality of life was assessed with the use of the European Organization for Research and Treatment of Cancer (EORTC) quality-of-life questionnaire for cancer (EORTC QLQ-C30) (28). The time of deterioration in the quality of life was calculated from enrollment to deterioration in the quality of life. And the deterioration is defined as a decrease of 10 points or more from baseline or death, whichever occurred first.

### Statistical analysis and sample size calculation

The prior sample size was calculated relative to the primary outcome achievement. All sample size calculations assumed an α of 0.05, under 2-sided hypothesis testing, and β error of 0.20 (power = 80%).

Our previous research showed that the 1-year RFS rates of high-risk patients were 52.7%. Assuming that combined anti-PD-1 adjuvant therapy, the 1-year RFS rates of high-risk patients can reach 75%. Meanwhile, assuming 24 months of planned enrollment and the longest follow-up period of 24 months, 21 subjects should be enrolled. Taking into account a dropout rate of 15%, 25 patients were required to detect this hypothesized reduction rate.

Continuous variables were expressed as mean (standard deviation [SD]) or median (IQR), while categorical variables were presented with frequency distributions (n, %). Comparisons between two groups were performed by using the independent-samples T-test, Mann-Whitney Wilcoxon test, or Pearson Chi-squared tests. RFS/OS were estimated by the Kaplan-Meier method and compared with log-rank tests. Univariate and multivariate analyses were conducted with Cox proportional hazards regression models to identify factors independently associated with RFS and OS. Subgroup analyses were performed by age, ECOG-PS, Child-Pugh score, CNLC staging, etiology, and tumor number. A Cox proportional hazards model was used to estimate the HR and 95% CIs for the group comparison.

To reduce selection bias and the effects of confounding factors, logistic regression was used to compute propensity scores and then matched the control group to the experimental group using a 1:1 ratio. Standardized mean differences below 0.2 indicate successful balance in the variables, including age, sex, Child-Pugh score, AFP, CNLC staging, ECOG-PS, recurrence risk grade, cirrhosis, ALT, and AST.

All statistical analyses were processed using SAS version9.4 software (SAS Institute, Cary, NC, USA) or R3.6.2 statistical software (R Foundation for Statistical Computing, Vienna, Austria). And all statistical tests were performed using a two-sided significance level of 0.05. In addition, the 95% confidence intervals and P values would be presented when calculating the difference in means between groups.

This analysis is a phased analysis of this trial. As of the data cut-off of December 31, 2021, a total of 15 patients were enrolled, of which 4 had relapsed. All data reported here are based on a phased analysis and have statistically meaningful results. The trial continues to accumulate long-term data.

## Results

### Baseline patient characteristics

**Figure 1:**
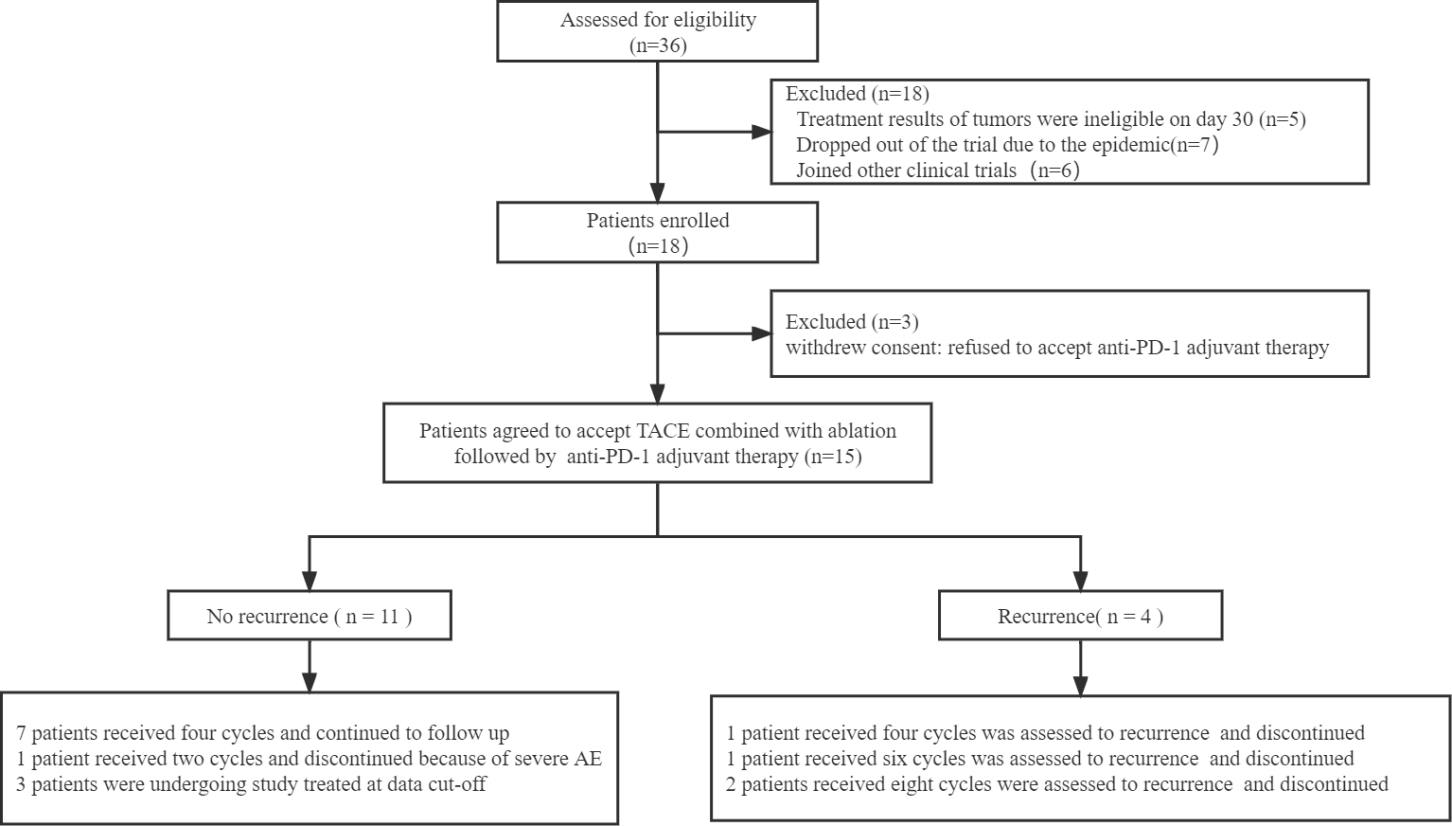
Flow chart of the patients included in the study. TACE: transcatheter arterial chemoembolization.

Among thirty-six patients who were screened between October 11, 2020, and December 31, 2021, 15 patients were enrolled and accepted combination therapy followed by anti-PD-1 adjuvant therapy (**Figure 1**). Of all the patients in the experimental group, 7 patients received radiofrequency ablation and 8 patients accepted microwave ablation. Then, one-to-one PSM produced 15 matched patient pairs who also received the combined treatment, containing 9 patients who underwent radiofrequency ablation and 6 patients treated with microwave ablation.

The baseline patient characteristics were similar between the two groups no significant differences were found in several important variables, such as age, gender, etiology, serum biochemical and AFP levels, Child-Pugh score, CNLC staging, ECOG-PS, tumor number, tumor size, recurrence risk grade and ablation modality (**Table 1**).

**Table 1:** Baseline characteristics of patients after propensity score analysis.

Variable	Experimental group (n=15)	Control group (n=15)	*P*-value
Age (IQR), year	59 (53–63)	60 (54-64)	0.718
Gender— no. (%)			1
male	15 (100%)	15 (100%)	
Etiology— no. (%)			0.135
HBV	13 (86.7%)	13 (86.7%)	
HCV	0	2 (13.3%)	
Alcohol	2 (13.3%)	0	
Cirrhosis— no. (%)	15 (100%)	15 (100%)	1
ECOG-PS— no. (%)			0.256
PS=0	7 (46.7%)	4 (26.7%)	
PS=1	8 (53.3%)	11 (73.3%)	
Child-Pugh score— no. (%)			1
A	12 (80%)	12 (80%)	
B	3 (20%)	3 (20%)	
CNLC staging— no. (%)			0.09
Ia	5 (33.3%)	3 (20%)	
Ib	7 (46.7%)	12 (80%)	
Ia or IIb	3 (20%)	0	
Ablative modality— no. (%)			0.464
RFA	7 (46.7%)	9 (60%)	
MWA	8 (53.3%)	6 (40%)	
Tumor number — no. (%)			0.136
Single	4 (26.7%)	8 (53.3%)	
Multiple	11 (73.3%)	7 (46.7%)	
Tumor size — no. (%)			0.409
≤2cm	10 (66.7%)	12 (80%)	
>2cm	5 (33.3%)	3 (20%)	
AFP (IQR), ng/mL	8.14 (3.01-20.9)	7.89 (3.38-30.7)	0.823
ALT (IQR), U/L	24 (15-29)	20 (13-30)	0.517
AST (IQR), U/L	26 (21-30)	28 (21-32)	0.651
TBIL (IQR), umol/L	15.6 (12.9-25.4)	20.1 (17-26.9)	0.345
PLT (IQR),10^9/L	138 (86-162)	104 (74-145)	0.187
ALB (IQR), g/L	38.1 (34.3-39.4)	40.4 (34.9-42)	0.285

HBV, hepatitis B virus; HCV, hepatitis C virus; ECOG-PS, Eastern Cooperative Oncology Group performance status; CNLC, China liver cancer staging RFA, radiofrequency ablation; MWA, microwave ablation; AFP, alpha-fetoprotein; ALT, alanine aminotransferase; AST, aspartate aminotransferase; TBIL, total bilirubin; PLT, platelet; ALB, albumin.

### Efficacy

**Figure 2:**
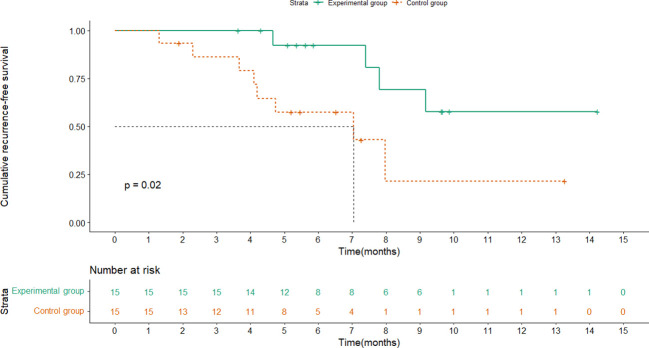
Kaplan-Meier plots for RFS in the experimental group and control group.

RFS was analyzed for the two groups, and the median follow-up time was 5.6 months(range,1.3-14.2). The median RFS (mRFS) of the control group was 7.0 months (95% CI: 2.0-12.0) while the experimental group had not reached it at the time of data cutoff. Moreover, the 1-year RFS rate was 73.3% (95% CI: 44.8%-91.1%) in the experimental group and 46.7% (95% CI: 22.2% - 72.6%) in the control group, showing a significant difference (HR, 0.251; 95% CI, 0.072-0.871; P =0.02) (**Figure 2**). As for OS, there were no treatment-related deaths in the two groups, and neither group reached the median OS. Note that no appreciable difference in prognosis was observed between patients treated for 4 cycles and 8 cycles(P=0.127).

The rate of LTP in the experimental group was 6.7% (95%CI: 3.5%-34.0%), which was clearly lower than that in the control group (40% with a 95%CI of 1%-75.9%, P = 0.027). And, the rate of IDR in experimental group and control group were 20% (95%CI: 5.3%-48.6%), 13.3% (95%CI: 2.3%-41.6%), respectively. EMs were not identified in either cohort (**Table 2**).

**Table 2:** Comparison of recurrence modality between the two groups.

Recurrence modality	Experimental groupEvents (%)	Control groupEvents (%)	Hazard ratio (95%CI)	P-value
LTP	1 (6.7)	6 (40)	0.088 (0.01-0.759)	**0.027**
IDR	3 (20)	2 (13.3)	0.722 (0.112-4.668)	0.733
EM	0	0	NE	NE

LTP, local tumor progression; IDR, intrahepatic distant recurrence; EM, extrahepatic metastasis; NE, not evaluated. The bolded values represent statistically significant differences.

**Figure 3:**
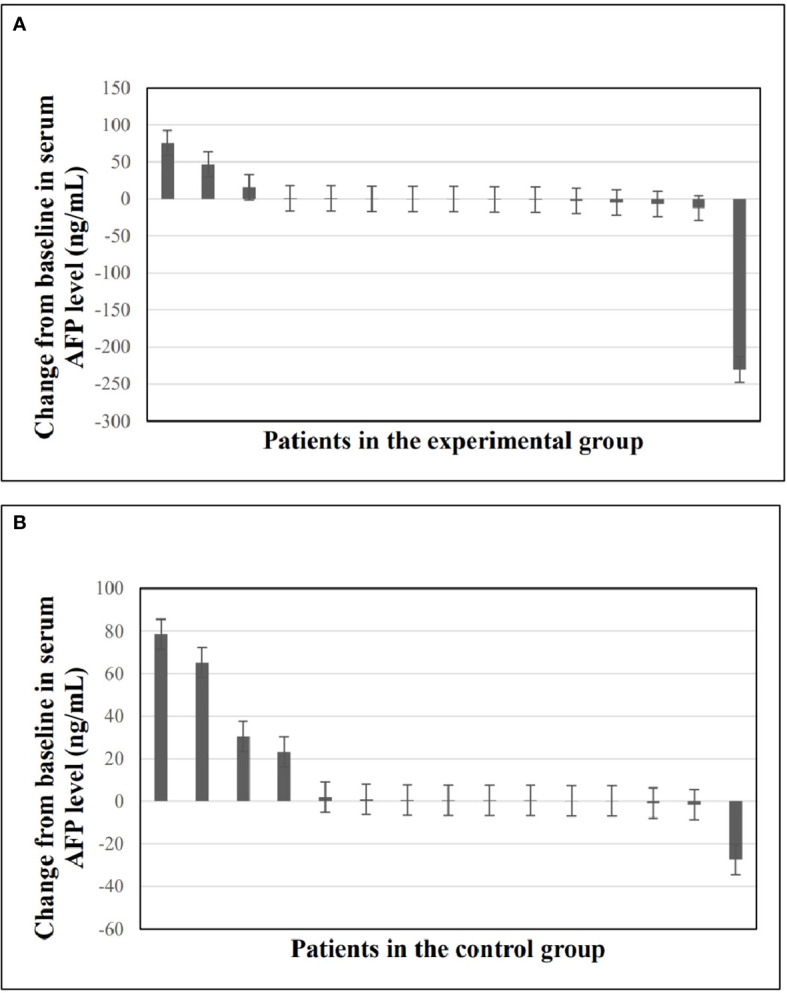
Changes in serum AFP levels from baseline to the last follow-up time. **(A)** Waterfall plot of changes in serum AFP levels in experimental group. **(B)** Waterfall plot of changes in serum AFP levels in control group. AFP, alpha-fetoprotein.

Waterfall plots were plotted to compare the change in serum AFP levels as a tumor marker from baseline to the last follow-up. As shown in **Figure 3**, serum AFP levels were decreased in 9 (60%) patients in the experimental group and 3 (20%) in the control group, with a statistically significant difference between the two groups (P=0.025).

Subgroup analyses showed that anti-PD-1 adjuvant therapy provided clinical benefits in all subgroups. In particular, benefits associated with anti-PD-1 adjuvant therapy were observed in patients with multiple tumors (HR =0.209, 95%CI: 0.048-0.91, P =0.037) and tumor size ≤2cm (HR =0.118,95%CI:0.014-1.0, P =0.05) (**Table 3**).

**Table 3:** Subgroup analysis for RFS according to patient characteristics at baseline.

Subgroup	Value	P-Value	HR (95%CI)
Age	<60	0.165	0.201 (0.021-1.937)
	≥60	0.067	0.128 (0.014-1.159)
ECOG-PS	PS=0	0.581	0.007 (0-263828.176)
	PS=1	0.145	0.394 (0.113-1.379)
Child-Pugh score	A	0.108	0.343 (0.093-1.265)
	B	0.471	0.015 (0-1327.44)
CNLC staging	Ia	0.434	0.007 (0-1792.569)
	Ib	0.157	0.304 (0.058-1.585)
Etiology	HBV	0.082	0.303 (0.079-1.163)
	Other	0.471	0.015 (0-1327.44)
Tumor number	Single	0.451	0.023 (0-406.493)
	Multiple	**0.037**	0.209 (0.048-0.91)
Tumor size	≤2cm	**0.05**	0.118 (0.014-1.0)
	>2cm	0.616	0.002 (0-47438567.93)

RFS, Recurrence-free survival; HR, hazard ratio; CI, confidence interval; ECOG-PS, Eastern Cooperative Oncology Group performance status; CNLC, China liver cancer staging. Notes: no subgroup analysis was performed for CNLC II a or II b patients due to no CNLC II a or II b patients in the control group. The bolded values represent statistically significant differences.

Prognostic factors affecting RFS were analyzed. Univariate and multivariate analyses including age, Child-Pugh score, CNLC staging, AFP level, and methods of therapy (combination therapy followed by anti-PD-1 adjuvant therapy versus combination therapy) were conducted in all study patients (N = 30). Results showed that anti-PD-1 adjuvant therapy (adjusted HR, 0.251; 95% CI, 0.072-0.871; P =0.029) was a significant favorable predictor of RFS (**Table 4**).

**Table 4:** Univariate and multivariate analysis of factors associated with RFS.

Variables	Univariate analysis		Multivariate analysis	
	HR (95%CI)	*P* value	HR (95%CI)	*P* value
Age	0.974 (0.883-1.074)	0.599		
Child-Pugh score	1.545 (0.304-7.86)	0.6		
CNLC staging	0.322 (0.38-2.734)	0.299		
AFP level	1.0 (0.983-1.017)	0.956		
Methods of therapy	0.150 (0.026-0.857)	**0.033**	0.251 (0.072-0.871)	**0.029**

CNLC, China liver cancer staging; AFP, alpha-fetoprotein. The bolded values represent statistically significant differences.

The quality of life of the patients in the experimental group was assessed when they received anti-PD-1 adjuvant therapy, while matched patients in the control group were followed up by telephone or outpatient to evaluate their quality of life. By December 31, 2021, all patients had completed quality-of-life assessments, and none of them experienced a deterioration in their quality of life.

### Safety

As for safety, data from 15 patients in the experimental group were analyzed. The rate of any grade of treatment-related adverse events (TRAE) was 60% (9/15), including 53.3% (8/15) for grades 1-2 AE, 6.7% (1/15) for grades≥3 AE, and 6.7% (1/15) for severe AE. One patient (6.7%) discontinued due to severe adverse events, and no treatment-related deaths occurred (**Table 5**).

**Table 5:** Adverse events related to anti-PD-1 treatment.

Variable	Experimental group (n=15)
Treatment-related adverse events.	9 (60%)
Grade 1–2 adverse events	8 (53.3%)
Adverse events of ≥grade 3	1 (6.7%)
Serious adverse events	1 (6.7%)
Serious adverse events leading to drug discontinuation	1 (6.7%)
Serious adverse events leading to death	0

The most frequently grade 1-2 AE were reactive cutaneous capillary endothelial proliferation (RCCEP) (20%), fatigue (13.3%), increased AST (13.3%), and increased ALT (13.3%). Some patients experienced adverse events such as a rash (6.7%), pruritus (6.7%), increased blood bilirubin (6.7%), decreased platelet count (6.7%), and diarrhea (6.7%). The AE of grade 3 or higher that occurred was hyperthyroidism (13.3%) (**Table 6**).

**Table 6:** Adverse events with an incidence of more than 5% in the experimental group.

Variable	Experimental group (n=15)	
	All Grade	≥ Grade 3
Hypothyroidism	0	0
Hyperthyroidism	2 (13.3%)	1 (6.7%)
Rash	1 (6.7%)	0
Increased aspartate aminotransferase	2 (13.3%)	0
Increased alanine aminotransferase	2 (13.3%)	0
Increased blood bilirubin	1 (6.7%)	0
Decreased platelet count	1 (6.7%)	0
RCCEP	3 (20%)	0
Pruritus	1 (6.7%)	0
Diarrhea	1 (6.7%)	0
Fatigue	2 (13.3%)	0
Fever	0	0
Infusion-related reaction	0	0

RCCEP, reactive cutaneous capillary endothelial proliferation.

## Discussion

The present study was the first clinical study based on the scoring system of recurrence in HCC patients to explore the efficacy and safety of anti-PD-1 adjuvant therapy after locoregional therapy in early-to-mid-stage HCC patients with a high risk of recurrence. Interim results showed that adjuvant anti-PD-1 therapy was an effective and tolerable treatment regimen with encouraging results in RFS, quality of life, safety, and rate of LTP.

There were reports that Sorafenib was not an effective intervention in the adjuvant setting for HCC following resection or ablation (29), while our study showed encouraging outcomes, which had many success factors. First of all, our previous experimental results confirmed that ablation affected T-cell immunity which plays a role in the occurrence and development of HCC (22, 30); and demonstrated that recurrence after ablation correlates with poor immune reconstitution, which may be influenced by activation of the PD-1 signaling pathway (unpublished data). Additionally, based on our scoring system, this study accurately screened patients at high risk of recurrence. In the era of precision medicine, choosing the optimal treatment strategy for individualized treatment will also achieve early prevention of disease recurrence.

In our study, anti-PD-1 adjuvant therapy was highly effective in reducing the risk of recurrence (HR=0.251). Compared with the control group, the risk of recurrence in the experimental group was reduced by 74.9%, suggesting that anti-PD-1 adjuvant therapy can provide significant clinical benefits for RFS which also was proved in other results of this study. First, AFP, as a single biomarker for diagnosing HCC, can well monitor treatment response (31, 32). **Figure 3** shows that anti-PD-1 adjuvant therapy can effectively reduce AFP in the experimental group, which could reflect the therapeutic effect of anti-PD-1 adjuvant therapy. Second, although it did not reach a significant difference in most subgroups, the benefit of anti-PD-1 adjuvant therapy showed a positive trend in RFS and could effectively decrease the relapse risk of patients with multiple tumors and tumor size ≤2cm to 79.1% and 89.2%, respectively. Finally, multivariate analysis showed that anti-PD-1 adjuvant therapy was an independent favorable prognostic factor for RFS in HCC patients.

Besides clinical factors such as tumor load or vascular invasion, studies have also proved that immunity mechanisms were related to the recurrence of HCC (33–38) The tumor immune microenvironment of the liver plays a crucial role in the recurrence of HCC after ablation. Ablation, especially radiofrequency ablation, could induce multiple mechanisms for immune protection. Because that thermal damage could promote tumor cells to release tumor antigens and upregulate inflammatory cytokines and cytotoxic T-cell subsets in the sublethal zone adjacent to the ablation (39). Relevant tumor-specific T-cell responses were enhanced within weeks after ablation, and the number of induced T-cells is associated with RFS (40). However, the effective immunogenicity induced by ablation is not sufficient to prevent a recurrence ultimately. PD-1 inhibitors could block the tumor immune escape pathway and maintain the T cells’ tumor cell-killing activity *via* binding to PD-1 on the surface of T cells (41, 42). As shown in **Figure 2**, there was a significant difference in RFS between the experimental and control groups, suggesting that combined immunotherapy after locoregional interventional therapy could enhance the anti-tumor immune response within the tumor microenvironment to reduce or prevent a recurrence, which may prove that anti-PD-1 adjuvant therapy can effectively improve the therapeutic effect, which is exactly as we expected. Based on the high-risk relapse population, local therapy combined with a systemic system, especially in combination with anti-PD-1 adjuvant therapy, should be well explored for improving the prognosis of HCC patients, proposing a promising approach for future combination therapy.

Compared with the control group, the LTP rate of the experimental group was significantly reduced. Our team’s data (22) showed that ablation therapy could improve the patient’s tumor-associated antigen (TAA)-specific T-cell immune response in the short term, accompanied by changes in PD1 expression levels. Simultaneously, PD-1 inhibitors further maintain T cell function by blocking the inhibitory signaling of PD-1 molecules on the surface of T cells (43). In addition, ablation could promote local cell infiltration and perifocal antigen release, resulting in T-cell activation (29). However, as the immune improvement caused by ablation is not lasting and is accompanied by tumor constitution; its control effect on distant lesions in the liver may not be significant (unpublished data). Further experiments are needed to determine such mechanisms.

All patients were alive by the date of the last follow-up, and longer-term follow-up is required to evaluate OS benefits associated with adjuvant anti-PD-1 therapy. At the same time, there was no deterioration in the quality of life in both groups, which may be because all patients underwent combination therapy to achieve complete recovery. Numerous studies have pointed out that TACE combined with Ablation in patients with early-stage HCC was superior to ablation alone, even in intermediate-stage HCC patients (44–47). Thus, all patients in this study received combination therapy.

The most frequent AE observed in this study were reactive cutaneous capillary endothelial proliferation (RCCEP, which is a specific AE of camrelizumab), and other AEs included gastrointestinal reactions (diarrhea), hepatotoxicity (changes in indicators of liver functions), skin reactions (rash and pruritus), and fatigue, which were consistent with the safety profile of anti-PD-1 therapy previously reported (48–50). None of the patients showed fever or infusion reactions. Several researchers reported that enhanced immune responses induced by immune checkpoint inhibitors could cause severe thyroid disorders (51–53). In our study, one patient withdrew from the trial due to severe hyperthyroidism. As the enrolled patients with early or intermediate‐stage liver cancer were in good physical condition to tolerate the toxicity caused by anti-PD-1 adjuvant therapy, the incidence of GRADE ≥3 AE and withdrawal in this study was low with no deaths recorded. Overall, anti-PD-1 adjuvant therapy was safe and well-tolerated for HCC patients in the early-intermediate stage.

Nevertheless, several limitations exist in the study. First of all, this study is a single-center study limited to an Asian population with small sample size. And it included a widely varied patient population from a Chinese institute, so selection bias is inevitable. However, the control group matched by PSM might somehow balance the selection bias, and the high RFS rate in the control group reflected these patients’ relapse risk. And our study also provides a promising idea to improve the prognosis for other populations with HCC. Another limitation of this study was that the total number of patients analyzed was relatively small, with a comparatively short follow-up period. Our previous studies showed that early recurrence often occurs in the first 2-3 years after ablation, making up about 70% of recurrence events (19). Moreover, as an interim analysis, our study is not concluded here. Follow-up will be continued and outcomes, including both relapse and long-term survival, will be reported in the future. Future studies with multicenter, large sample sizes, and long follow-up duration will be needed.

## Conclusion

In summary, interim data suggest the addition of anti-PD-1 adjuvant therapy after TACE combined with ablation can significantly prolong RFS with controllable safety for early-to-mid-stage HCC patients. In a more general precision medicine context, screening high-risk recurrence patients for combination therapy can effectively augment the outcome of patients’ prognosis and long-term survival quality, gaining important clinical significance. Therefore, the combination of therapeutic regimens could inform novel treatment options for this patient population.

## Data availability statement

The raw data supporting the conclusions of this article will be made available by the authors, without undue reservation.

## Ethics statement

The studies involving human participants were reviewed and approved by Ethics Committee of Capital Medical University affiliated Beijing You’an Hospital. The patients/participants provided their written informed consent to participate in this study.

## Author contributions

Conceived and designed the protocol: ZYH; Collected data: SY and LBY; Analyzed data: QWY and WW; Wrote the manuscript: WQ and QWY; Critically revised and approved the final version of manuscript: LJJ and YCW; Treated and observed the patients: ZYH and HCX; All authors contributed to the article and approved the submitted version.

## Funding

This study was funded by a grant Beijing Municipal Natural Science Foundation (7202069 and 7191004), Capital health development project (CFH2020-1-2182 and CFH2020-2-1153), Beijing Key Laboratory (BZ0373), Beijing Municipal Administration of Hospitals’ Ascent Plan (DFL20181701), Beijing Municipal Science & Technology Commission (Z171100001017078), Key medical professional development plan of Beijing municipal administration of hospitals (ZYLX201711), Beijing Incubating Program (PX2018059 and PX2022067), and Beijing Municipal Administration of Hospitals’ Youth Programmer (QML20211709).

## Acknowledgments

The authors would like to thank Innovent Biologics (Suzhou) Co., Ltd. and Jiangsu Hengrui Pharmaceuticals Co., Ltd. for providing the free drugs (Sintilimab and Camrelizumab).

## Conflict of interest

The authors declare that the research was conducted in the absence of any commercial or financial relationships that could be construed as a potential conflict of interest.

## Publisher’s note

All claims expressed in this article are solely those of the authors and do not necessarily represent those of their affiliated organizations, or those of the publisher, the editors and the reviewers. Any product that may be evaluated in this article, or claim that may be made by its manufacturer, is not guaranteed or endorsed by the publisher.

## References

[1] Sung H Ferlay J Siegel RL Laversanne M Soerjomataram I Jemal A. Global cancer statistics 2020: Globocan estimates of incidence and mortality worldwide for 36 cancers in 185 countries. CA: Cancer J Clin (2021) 71(3):209–49. doi: 10.3322/caac.21660 33538338

[2] Couri T Pillai A. Goals and targets for personalized therapy for hcc. Hepatol Int (2019) 13(2):125–37. doi: 10.1007/s12072-018-9919-1 30600478

[3] Raoul JL Forner A Bolondi L Cheung TT Kloeckner R de Baere T. Updated use of tace for hepatocellular carcinoma treatment: How and when to use it based on clinical evidence. Cancer Treat Rev (2019) 72:28–36. doi: 10.1016/j.ctrv.2018.11.002 30447470

[4] Yang JD Hainaut P Gores GJ Amadou A Plymoth A Roberts LR. A global view of hepatocellular carcinoma: Trends, risk, prevention and management. Nat Rev Gastroenterol Hepatol (2019) 16(10):589–604. doi: 10.1038/s41575-019-0186-y 31439937 PMC6813818

[5] Livraghi T Meloni F Di Stasi M Rolle E Solbiati L Tinelli C. Sustained complete response and complications rates after radiofrequency ablation of very early hepatocellular carcinoma in cirrhosis: Is resection still the treatment of choice? Hepatol (Baltimore Md) (2008) 47(1):82–9. doi: 10.1002/hep.21933 18008357

[6] Forner A Reig M Bruix J. Hepatocellular Carcinoma. Lancet (London, England) (2018) 39(1027):1301–14. doi: 10.1016/s0140-6736(18)30010-2 29307467

[7] De Lorenzo S Tovoli F Barbera MA Garuti F Palloni A Frega G. Metronomic capecitabine vs. best supportive care in child-pugh b hepatocellular carcinoma: A proof of concept. Sci Rep (2018) 8(1):9997. doi: 10.1038/s41598-018-28337-6 29968763 PMC6030080

[8] Rizzo A Nannini M Novelli M Dalia Ricci A Scioscio VD Pantaleo MA. Dose reduction and discontinuation of standard-dose regorafenib associated with adverse drug events in cancer patients: A systematic review and meta-analysis. Ther Adv Med Oncol (2020) 12:1758835920936932. doi: 10.1177/1758835920936932 32684988 PMC7343359

[9] Rizzo A Ricci AD Di Federico A Frega G Palloni A Tavolari S. Predictive biomarkers for checkpoint inhibitor-based immunotherapy in hepatocellular carcinoma: Where do we stand? Front Oncol (2021) 11:803133. doi: 10.3389/fonc.2021.803133 34976841 PMC8718608

[10] Rizzo A Ricci AD Gadaleta-Caldarola G Brandi G. First-line immune checkpoint inhibitor-based combinations in unresectable hepatocellular carcinoma: Current management and future challenges. Expert Rev Gastroenterol Hepatol (2021) 15(11):1245–51. doi: 10.1080/17474124.2021.1973431 34431725

[11] Huang J Huang W Guo Y Cai M Zhou J Lin L. Risk factors, patterns, and long-term survival of recurrence after radiofrequency ablation with or without transarterial chemoembolization for hepatocellular carcinoma. Front Oncol (2021) 11:638428. doi: 10.3389/fonc.2021.638428 34123790 PMC8191459

[12] Preel A Hermida M Allimant C Assenat E Guillot C Gozzo C. Uni-, bi- or trifocal hepatocellular carcinoma in Western patients: Recurrence and survival after percutaneous thermal ablation. Cancers (2021) 13(11):2700. doi: 10.3390/cancers13112700 34070800 PMC8197823

[13] Yi PS Huang M Zhang M Xu L Xu MQ. Comparison of transarterial chemoembolization combined with radiofrequency ablation therapy versus surgical resection for early hepatocellular carcinoma. Am surgeon (2018) 84(2):282–8. doi: 10.1177/000313481808400238 29580359

[14] Fujiwara N Friedman SL Goossens N Hoshida Y. Risk factors and prevention of hepatocellular carcinoma in the era of precision medicine. J Hepatol (2018) 68(3):526–49. doi: 10.1016/j.jhep.2017.09.016 PMC5818315 28989095

[15] Liang BY Gu J Xiong M Zhang EL Zhang ZY Chen XP. Tumor size may influence the prognosis of solitary hepatocellular carcinoma patients with cirrhosis and without macrovascular invasion after hepatectomy. Sci Rep (2021) 11(1):16343. doi: 10.1038/s41598-021-95835-5 34381132 PMC8357938

[16] Reig M Forner A Rimola J Ferrer-Fàbrega J Burrel M Garcia-Criado Á. Bclc strategy for prognosis prediction and treatment recommendation: The 2022 update. J Hepatol (2022) 76(3):681–93. doi: 10.1016/j.jhep.2021.11.018 PMC8866082 34801630

[17] Sun X Mei J Lin W Yang Z Peng W Chen J. Reductions in afp and pivka-ii can predict the efficiency of anti-Pd-1 immunotherapy in hcc patients. BMC Cancer (2021) 21(1):775. doi: 10.1186/s12885-021-08428-w 34218801 PMC8254996

[18] Zhou P Chen B Miao XY Zhou JJ Xiong L Wen Y. Comparison of fib-4 index and child-pugh score in predicting the outcome of hepatic resection for hepatocellular carcinoma. J Gastrointestinal Surgery: Off J Soc Surg Alimentary Tract (2020) 24(4):823–31. doi: 10.1007/s11605-019-04123-1 31066014

[19] Wang Q Ma L Li J Yuan C Sun J Li K. A novel scoring system for patients with recurrence of hepatocellular carcinoma after undergoing minimal invasive therapies. Cancer Manage Res (2019) 11:10641–9. doi: 10.2147/cmar.S224711 PMC6930388 31908536

[20] Rich NE Yopp AC Singal AG. Medical management of hepatocellular carcinoma. J Oncol Pract (2017) 13(6):356–64. doi: 10.1200/jop.2017.022996 28605614

[21] Ribas A. T Cells as the future of cancer therapy. Cancer Discov (2021) 11(4):798–800. doi: 10.1158/2159-8290.Cd-21-0022 33811114 PMC8082738

[22] Zang C Zhao Y Qin L Liu G Sun J Li K. Distinct tumour antigen-specific T-cell immune response profiles at different hepatocellular carcinoma stages. BMC Cancer (2021) 21(1):1007. doi: 10.1186/s12885-021-08720-9 34496797 PMC8428121

[23] Prieto J Melero I Sangro B. Immunological landscape and immunotherapy of hepatocellular carcinoma. Nat Rev Gastroenterol Hepatol (2015) 12(12):681–700. doi: 10.1038/nrgastro.2015.173 26484443

[24] Pahwa A Beckett K Channual S Tan N Lu DS Raman SS. Efficacy of the American association for the study of liver disease and Barcelona criteria for the diagnosis of hepatocellular carcinoma. Abdominal Imaging (2014) 39(4):753–60. doi: 10.1007/s00261-014-0118-9 24699935

[25] Nishikawa H Inuzuka T Takeda H Nakajima J Sakamoto A Henmi S. Percutaneous radiofrequency ablation therapy for hepatocellular carcinoma: A proposed new grading system for the ablative margin and prediction of local tumor progression and its validation. J Gastroenterol (2011) 46(12):1418–26. doi: 10.1007/s00535-011-0452-4 21845378

[26] Nakazawa T Kokubu S Shibuya A Ono K Watanabe M Hidaka H. Radiofrequency ablation of hepatocellular carcinoma: Correlation between local tumor progression after ablation and ablative margin. AJR Am J Roentgenol (2007) 188(2):480–8. doi: 10.2214/ajr.05.2079 17242258

[27] Ahmed M Solbiati L Brace CL Breen DJ Callstrom MR Charboneau JW. Image-guided tumor ablation: Standardization of terminology and reporting criteria–a 10-year update. Radiology (2014) 273(1):241–60. doi: 10.1148/radiol.14132958 PMC4263618 24927329

[28] Nolte S Liegl G Petersen MA Aaronson NK Costantini A Fayers PM. General population normative data for the eortc qlq-C30 health-related quality of life questionnaire based on 15,386 persons across 13 European countries, Canada and the unites states. Eur J Cancer (Oxford England: 1990) (2019) 107:153–63. doi: 10.1016/j.ejca.2018.11.024 30576971

[29] Dromi SA Walsh MP Herby S Traughber B Xie J Sharma KV. Radiofrequency ablation induces antigen-presenting cell infiltration and amplification of weak tumor-induced immunity. Radiology (2009) 251(1):58–66. doi: 10.1148/radiol.2511072175 19251937 PMC2663580

[30] Zhao Y Li K Sun J He N Zhao P Zang C. Genomic DNA methylation profiling indicates immune response following thermal ablation treatment for hbv-associated hepatocellular carcinoma. Oncol Lett (2020) 20(1):677–84. doi: 10.3892/ol.2020.11636 PMC7285841 32565992

[31] Park SJ Jang JY Jeong SW Cho YK Lee SH Kim SG. Usefulness of afp, afp-L3, and pivka-ii, and their combinations in diagnosing hepatocellular carcinoma. Medicine (2017) 96(11):e5811. doi: 10.1097/md.0000000000005811 28296720 PMC5369875

[32] Yamamoto K Imamura H Matsuyama Y Kume Y Ikeda H Norman GL. Afp, afp-L3, dcp, and Gp73 as markers for monitoring treatment response and recurrence and as surrogate markers of clinicopathological variables of hcc. J Gastroenterol (2010) 45(12):1272–82. doi: 10.1007/s00535-010-0278-5 20625772

[33] Foerster F Hess M Gerhold-Ay A Marquardt JU Becker D Galle PR. The immune contexture of hepatocellular carcinoma predicts clinical outcome. Sci Rep (2018) 8(1):5351. doi: 10.1038/s41598-018-21937-2 29599491 PMC5876395

[34] Gabrielson A Wu Y Wang H Jiang J Kallakury B Gatalica Z. Intratumoral Cd3 and Cd8 T-cell densities associated with relapse-free survival in hcc. Cancer Immunol Res (2016) 4(5):419–30. doi: 10.1158/2326-6066.Cir-15-0110 PMC5303359 26968206

[35] Gao XH Tian L Wu J Ma XL Zhang CY Zhou Y. Circulating Cd14(+) hla-Dr(-/Low) myeloid-derived suppressor cells predicted early recurrence of hepatocellular carcinoma after surgery. Hepatol Research: Off J Japan Soc Hepatol (2017) 47(10):1061–71. doi: 10.1111/hepr.12831 27764536

[36] Liu GM Li XG Zhang YM. Prognostic role of pd-L1 for hcc patients after potentially curative resection: A meta-analysis. Cancer Cell Int (2019) 19:22. doi: 10.1186/s12935-019-0738-9 30718977 PMC6352338

[37] Shi F Shi M Zeng Z Qi RZ Liu ZW Zhang JY. Pd-1 and pd-L1 upregulation promotes Cd8(+) T-cell apoptosis and postoperative recurrence in hepatocellular carcinoma patients. Int J Cancer (2011) 128(4):887–96. doi: 10.1002/ijc.25397 20473887

[38] Zhu XD Zhang JB Zhuang PY Zhu HG Zhang W Xiong YQ. High expression of macrophage colony-stimulating factor in peritumoral liver tissue is associated with poor survival after curative resection of hepatocellular carcinoma. J Clin Oncol: Off J Am Soc Clin Oncol (2008) 26(16):2707–16. doi: 10.1200/jco.2007.15.6521 18509183

[39] Li G Staveley-O’Carroll KF Kimchi ET. Potential of radiofrequency ablation in combination with immunotherapy in the treatment of hepatocellular carcinoma. J Clin trials (2016) 6(2):257. doi: 10.4172/2167-0870.1000257 28042519 PMC5201112

[40] Mizukoshi E Yamashita T Arai K Sunagozaka H Ueda T Arihara F. Enhancement of tumor-associated antigen-specific T cell responses by radiofrequency ablation of hepatocellular carcinoma. Hepatol (Baltimore Md) (2013) 57(4):1448–57. doi: 10.1002/hep.26153 23174905

[41] El-Khoueiry AB Sangro B Yau T Crocenzi TS Kudo M Hsu C. Nivolumab in patients with advanced hepatocellular carcinoma (Checkmate 040): An open-label, non-comparative, phase 1/2 dose escalation and expansion trial. Lancet (London England) (2017) 389(10088):2492–502. doi: 10.1016/s0140-6736(17)31046-2 PMC7539326 28434648

[42] Zhu AX Finn RS Edeline J Cattan S Ogasawara S Palmer D. Pembrolizumab in patients with advanced hepatocellular carcinoma previously treated with sorafenib (Keynote-224): A non-randomised, open-label phase 2 trial. Lancet Oncol (2018) 19(7):940–52. doi: 10.1016/s1470-2045(18)30351-6 29875066

[43] Pardoll DM. The blockade of immune checkpoints in cancer immunotherapy. Nat Rev Cancer (2012) 12(4):252–64. doi: 10.1038/nrc3239 PMC4856023 22437870

[44] Endo K Kuroda H Oikawa T Okada Y Fujiwara Y Abe T. Efficacy of combination therapy with transcatheter arterial chemoembolization and radiofrequency ablation for intermediate-stage hepatocellular carcinoma. Scandinavian J Gastroenterol (2018) 53(12):1575–83. doi: 10.1080/00365521.2018.1548645 30577723

[45] Peng ZW Zhang YJ Liang HH Lin XJ Guo RP Chen MS. Recurrent hepatocellular carcinoma treated with sequential transcatheter arterial chemoembolization and Rf ablation versus Rf ablation alone: A prospective randomized trial. Radiology (2012) 262(2):689–700. doi: 10.1148/radiol.11110637 22157201

[46] Wang C Liao Y Qiu J Yuan Y Zhang Y Li K. Transcatheter arterial chemoembolization alone or combined with ablation for recurrent intermediate-stage hepatocellular carcinoma: A propensity score matching study. J Cancer Res Clin Oncol (2020) 146(10):2669–80. doi: 10.1007/s00432-020-03254-2 PMC11804750 32449005

[47] Zhang YJ Chen MS Chen Y Lau WY Peng Z. Long-term outcomes of transcatheter arterial chemoembolization combined with radiofrequency ablation as an initial treatment for early-stage hepatocellular carcinoma. JAMA Network Open (2021) 4(9):e2126992. doi: 10.1001/jamanetworkopen.2021.26992 34570206 PMC8477266

[48] Jiang FE Zhang HJ Yu CY Liu AN. Efficacy and safety of regorafenib or fruquintinib plus camrelizumab in patients with microsatellite stable and/or proficient mismatch repair metastatic colorectal cancer: An observational pilot study. Neoplasma (2021) 68(4):861–6. doi: 10.4149/neo_2021_201228N1415 33998237

[49] Wang F Qin S Sun X Ren Z Meng Z Chen Z. Reactive cutaneous capillary endothelial proliferation in advanced hepatocellular carcinoma patients treated with camrelizumab: Data derived from a multicenter phase 2 trial. J Hematol Oncol (2020) 13(1):47. doi: 10.1186/s13045-020-00886-2 32393323 PMC7216554

[50] Wang Y Zhou S Yang F Qi X Wang X Guan X. Treatment-related adverse events of pd-1 and pd-L1 inhibitors in clinical trials: A systematic review and meta-analysis. JAMA Oncol (2019) 5(7):1008–19. doi: 10.1001/jamaoncol.2019.0393 PMC6487913 31021376

[51] Ferrari SM Fallahi P Galetta F Citi E Benvenga S Antonelli A. Thyroid disorders induced by checkpoint inhibitors. Rev endocr Metab Disord (2018) 19(4):325–33. doi: 10.1007/s11154-018-9463-2 30242549

[52] Jannin A Penel N Ladsous M Vantyghem MC Do Cao C. Tyrosine kinase inhibitors and immune checkpoint inhibitors-induced thyroid disorders. Crit Rev Oncol/Hematol (2019) 141:23–35. doi: 10.1016/j.critrevonc.2019.05.015 31202955

[53] Wright JJ Powers AC Johnson DB. Endocrine toxicities of immune checkpoint inhibitors. Nat Rev Endocrinol (2021) 17(7):389–99. doi: 10.1038/s41574-021-00484-3 PMC8769055 33875857

